# Erythrocyte Indices in Creutzfeldt–Jakob Disease Predict Survival Time

**DOI:** 10.3389/fneur.2022.839081

**Published:** 2022-02-14

**Authors:** Yu Kong, Zhongyun Chen, Jing Zhang, Liyong Wu

**Affiliations:** Department of Neurology, Xuanwu Hospital, Capital Medical University, Beijing, China

**Keywords:** Creutzfeldt-Jakob disease, survival time, hemoglobin, hematocrit, erythrocyte indices, red cell indices

## Abstract

**Background:**

Creutzfeldt–Jakob disease (CJD) is a devastating neurodegenerative disease caused by propagation of abnormally folded prion proteins (PrP^Sc^). Some fluid biomarkers have been reported to be associated with disease duration in CJD. Based on studies which have found that prion protein (PrP^C^) played a role in erythrocytic hematopoiesis, we evaluated the association between peripheral red blood cell indices and survival time in CJD.

**Methods:**

We retrospectively collected data on peripheral red blood cell indices, including red blood cell (RBC) count, hemoglobin (Hb), hematocrit (HCT), mean corpuscular volume (MCV), mean corpuscular hemoglobin (MCH), mean corpuscular hemoglobin concentration (MCHC), and red cell distribution width (RDW), from 125 CJD patients. Cox proportional hazard models were generated to determine whether red cell indices correlated with survival time of patients with CJD.

**Results:**

Of the 125 included participants, 70 (56%) were male, and the mean age at diagnosis (SD) was 60.3 (9.5) years. Hemoglobin levels (hazard ratio 1.710, 95% CI 1.124–2.600, *p* = 0.012) and HCT (hazard ratio 1.689, 95% CI 1.112–2.565, *p*=0.014) were significantly associated with survival time after controlling for sex, age, and Barthel Index. Red blood cell count, MCV, MCH, MCHC, and RDW were not associated with survival time before or after adjusting for covariates.

**Conclusions:**

Our study found that Hb and HCT were significantly associated with survival time in patients with CJD. These results may inform evaluation of the mechanisms of interaction between prion disease and hematopoiesis, and indicate that Hb and HCT may be potential prognostic biomarkers.

## Introduction

Creutzfeldt-Jakob disease (CJD) is a rapidly progressive and fatal neurodegenerative disease caused by the propagation of abnormally folded prion proteins (PrP^Sc^) (1). The survival time of patients with CJD is variable, and ranges from weeks to several years, with a typical duration of 4 to 6 months (2–5). The speed at which CJD progresses and the fact that it is incurable highlights the importance of prediction of disease duration for disease management and clinical trial design. Several studies have found that several fluid biomarkers, such as cerebrospinal fluid (CSF) and plasma total tau (t-tau) levels, neurofilament light (NFL), the ratio of t-tau to phosphorylated tau (p-tau), and the 14-3-3 test result, were predictive of disease duration (3, 6–10). However, these biomarkers are relatively difficult to measure in some areas due to cost and technical limitations. Therefore, it is necessary to find a biomarker that is easy to measure.

Measurement of red cell indices is cost-effective and routine, and these indices have been shown to be associated with disease progression in some neurodegenerative diseases such as Alzheimer's disease (AD). However, red cell indices have not been evaluated as biomarkers for CJD (11). The expression of PrP^C^ on circulating red blood cells (RBCs) and the role of erythroid differentiation in CJD have been reported, which indicates that prions may play a role in hematopoiesis (12–14). The downregulation of erythroid genes and significantly decreased transcript levels of α-hemoglobin stabilizing protein (AHSP) in prion-infected animals also demonstrated a connection between prion disease and peripheral erythropoiesis (15, 16). Based on previous studies, we hypothesized that peripheral blood erythrocyte indices may predict disease course in patients with CJD. Therefore, we investigated the association between red cell indices and survival time.

## Methods

### Ethics Statement

This study was approved by the Ethics Committee of Xuanwu Hospital, Capital Medical University. All participants and/or their legal guardians provided written informed consent before undergoing any study procedures.

### Participants

**Figure 1:**
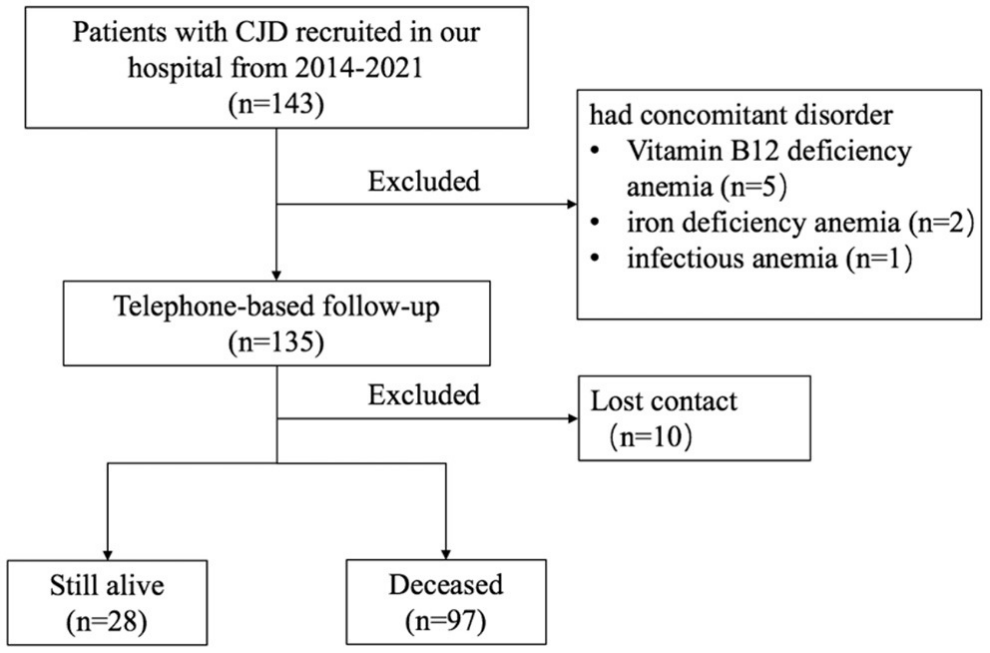
Flow diagram of patient enrollment.

Participants were referred to the Department of Neurology, Xuanwu Hospital, Capital Medical University, from 2014 to April 2021. In total, 143 patients diagnosed with probable and definite (confirmed genetically) CJD based on standard diagnostic criteria were enrolled (17). Eight patients who had comorbid disorders that could potentially affect erythroid hematopoietic function, or other blood disorders, were excluded from this study. Telephone-based follow-ups were conducted by neurologists from our hospital to assess survival time. By the end of October 2021, 97 out of the 135 enrolled patients were deceased, 28 were living, and 10 were unable to be contacted (Figure 1). Survival time was defined as the time from onset of first symptom to death.

### Collection of Clinical Data

Clinical data were collected for all enrolled patients. Electroencephalography (EEG), magnetic resonance imaging (MRI), and lumbar puncture were performed during hospitalization. All participants were administered the Barthel Index, a measure of functional severity commonly used to evaluate prion diseases (18, 19). MRI scans were performed on a 3.0 Tesla MRI system (Siemens Magnetom Trio Tim MRI system, Germany) using standard coil. T1-weighted, T2-weighted, fluid-attenuated inversion recovery (FLAIR), diffusion-weighted image (DWI), apparent diffusion coefficient (ADC) data were acquired. EEG monitoring was performed using a 32-channel digital EEG system (DAVINCI-SAM, Micromed, Mogliano Veneto, Italy). Cerebrospinal fluid (CSF) and blood samples were collected by the medical staff in our hospital and transferred to the Chinese Center for Disease Control and Prevention, where CSF 14-3-3 protein was detected by western blot and the prion protein gene (*PRNP*) was analyzed (20). Typical MRI imaging for CJD diagnosis was defined as the high signal of DWI or FLAIR in caudate/putamen or at least two cortical regions (temporal, parietal, occipital). Typical EEG pattern was defined as periodic sharp wave complexes (PSWCs) (17).

### Blood Sampling and Measurement of Blood Indices

Blood samples from participants were collected between 6:00 a.m. and 7:00 a.m. and sent to the hospital lab for testing within one hour. Red blood cell indices included red blood cell (RBC) count, hemoglobin (Hb), hematocrit (HCT), mean corpuscular volume (MCV), mean corpuscular hemoglobin (MCH), mean corpuscular hemoglobin concentration (MCHC), and red cell distribution width (RDW). In the case of blood samples collected at multiple time points after admission to the hospital, we used the first sample collected.

### Western Blot for 14-3-3 Protein in CSF

A total of 20μl CSF sample was separated by 12% SDS-PAGE and electronically transformed onto nitrocellulose membrane (Whatman, USA). Blots were incubated in 1:1000 diluted 14-3-3 polyclonal antibodies (Santa Cruz, USA) and further incubated in 1:5000 diluted HRP-conjugated goat anti-Rabbit IgG (PerkinElmer, Germany). Reactive signals were visualized using an enhanced chemiluminescence kit (Amersham-Pharmacia Biotech, USA). Each test contained a preparation of goat brain tissue homogenate as the positive control. The 14-3-3 test was considered positive only when the 14-3-3 immunoreactivity was comparable with that of the positive control (21).

### Statistical Analysis

Continuous variables are presented as the mean ± standard deviation (SD) or median and interquartile range (IQR). Categorical data are presented as the frequency (percentage). Spearman's rho correlation analysis was used to determine the associations between biochemical variables and survival time, and biochemical variables and Barthel Index. Mann-Whitney U test was performed to compare the erythrocyte indices between 14-3-3-positive patients and 14-3-3-negative patients. All red cell indices were binned in the median. Cox proportional hazard models were used to predict survival time. Three covariates (sex, age, Barthel Index) were fitted in three separate Cox proportional hazard models. All red cell indices were fitted in separate Cox proportional hazard models with and without covariates. A two-sided *p* value < 0.05 was considered statistically significance. All statistical analyses were performed using SPSS, version 25.

## Results

### Baseline Characteristics of the Sample

A total of 125 patients with CJD were enrolled in this study, of whom 70 (56.0%) were male. The mean age at diagnosis (SD) was 60.3 (9.5) years. Five of 125 patients were diagnosed with definite CJD (confirmed genetically), while 120 patients were diagnosed with probable CJD. Ninety-seven (77.6%) patients were deceased at the time of data collection. Detailed demographic and clinical characteristics are shown in Table 1.

**Table 1:** Participant demographic and clinical characteristics.

**Characteristic**	**No. (%)**
Total, No.	125
Age at study visit, years	
Mean (SD)	60.3 (9.5)
Median (IQR)	61.0 (56.0–65.5)
Range	30.0–82.0
Male/Female	70/55
Creutzfeldt–Jakob disease	
Genetically confirmed	5 (4.0%)
Probable	120 (96.0%)
Barthel Index score at first visit	
Mean (SD)	51.8 (28.3)
Median (IQR)	60.0 (30.0–75.0)
Range	0–95.0
A typical EEG, positive/total	50 (41.7%)/120
CSF 14-3-3 (+)	48 (44.0%)/109
A typical MRI brain scan, positive/total	112 (90.3%)/124
Deceased	97 (77.6%)
Average time from first symptom to death, months	
Mean (SD)	14.3 (10.7)
Median (IQR)	12.0 (5–21.5)
Range	2.0–51.0
< =6 months	34 (35.1%)
< =12 months	54 (55.7%)
< =24 months	77 (79.4%)

**Figure 2:**
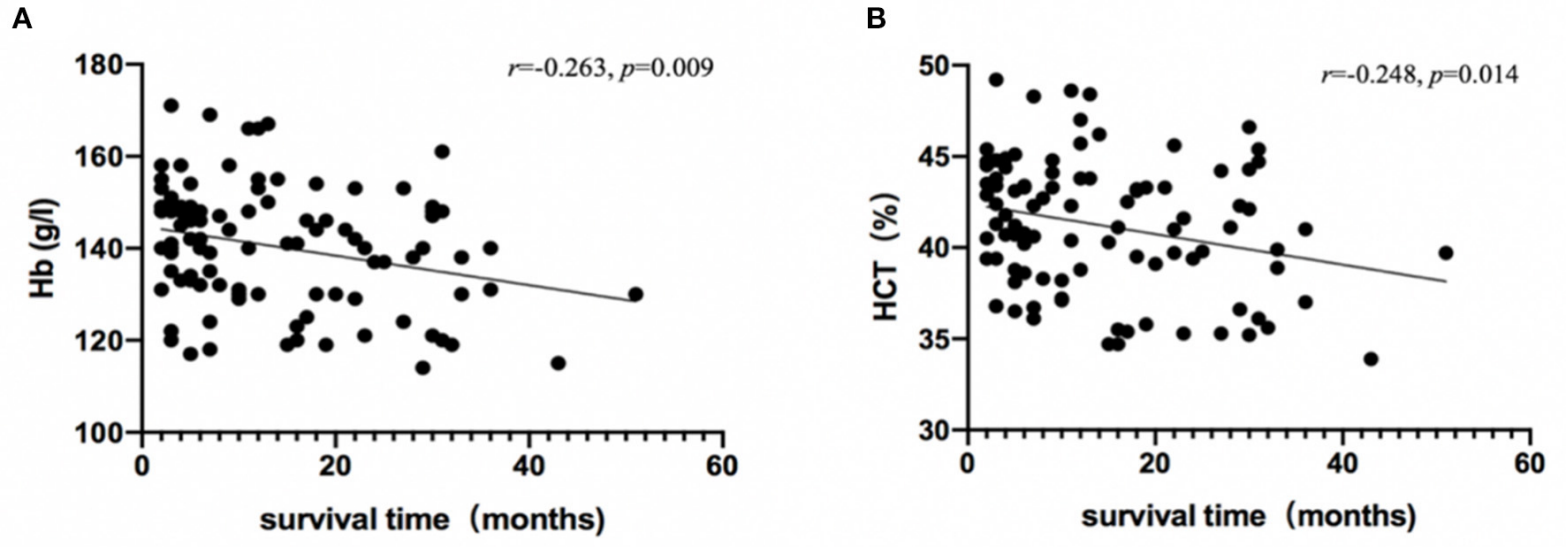
Association between survival time and red cell indices. Hb **(A)** and HCT **(B)** levels in patients with CJD are plotted against survival time, and were analyzed using Spearman's rho correlation analysis.

Hemoglobin (*r* = −0.263, *p* = 0.009) and HCT (*r* = −0.248, *p* = 0.014) were significantly associated with survival time (Figure 2), and no red cell indices correlated with functional impairment level (measured by Barthel Index). Descriptive statistics for red cell index values are presented in Table 2. In addition, we analyzed the relationship between erythrocyte indices and 14-3-3 protein. No difference between 14-3-3-positive group and 14-3-3-negative group (data not shown) was found.

**Table 2:** Red cell indices and cohort characteristics of deceased patients.

**Red cell index**	**Sample,** **total No**.	**Median (IQR)** **[range]**	**Correlation with Barthel index, *r***	***p* value**	**Correlation with survival time, *r***	***p* value**
RBC	97	4.52 (4.19, 4.83) [3.11, 5.42]	0.168	0.101	−0.192	0.059
Hb	97	141.00 (130.00–149.00) [114.00–171.00]	0.127	0.215	−0.263	0.009
HCT	97	41.20 (38.70–43.95) [33.90–49.20]	0.171	0.095	−0.248	0.014
MCV	97	91.20 (88.70–94.00) [71.90, 113.50]	−0.007	0.946	−0.058	0.572
MCH	97	31.20 (30.20–32.05) [23.00–39.90]	−0.084	0.415	−0.105	0.307
MCHC	97	342.00 (332.50–348.00) [311.00–358.00]	−0.051	0.621	−0.037	0.716
RDW	97	12.70 (12.30–13.10) [10.10–16.80]	−0.179	0.079	−0.129	0.209

### Variables Associated With Survival

We first evaluated the associations of age, sex, and Barthel Index with survival. Age (HR 1.513, 95% CI 1.006–2.276, *p* = 0.047) and Barthel Index (HR 0.545, 0.354–0.839, *p* = 0.006), but not sex, were significantly associated with survival time (Table 3). Younger age and higher baseline levels of function predicted longer disease duration. Interestingly, when both Barthel Index and age were entered simultaneously as predictors of survival, only Barthel Index remained statistically significant (HR 0.575, 95% CI 0.371–0.891, *p* = 0.013).

**Table 3:** Cox proportional hazard models of the associations between red cell indices and survival.

	**No Covariates**			**Covariates**		
	**HR**	**95% CI**	***p* Value**	**HR**	**95% CI**	***p* Value**
Covariate						
Age	1.513	1.006–2.276	0.047	NA	NA	NA
Sex	1.115	0.739–1.682	0.604	NA	NA	NA
Barthel Index	0.545	0.354–0.839	0.006	NA	NA	NA
Red cell Index						
RBC	1.240	0.827–1.860	0.298	1.374	0.901–2.096	0.141
Hb	1.710	1.124–2.600	0.012	1.959	1.232–3.116	0.004
HCT	1.689	1.112–2.565	0.014	1.927	1.234–3.010	0.004
MCV	0.857	0.572–1.284	0.454	0.788	0.520–1.195	0.262
MCH	1.228	0.822–1.836	0.316	1.133	0.749–1.716	0.554
MCHC	1.460	0.972–2.193	0.068	1.504	0.973–2.326	0.066
RDW	0.999	0.664–1.502	0.995	0.954	0.633–1.439	0.824

**Figure 3:**
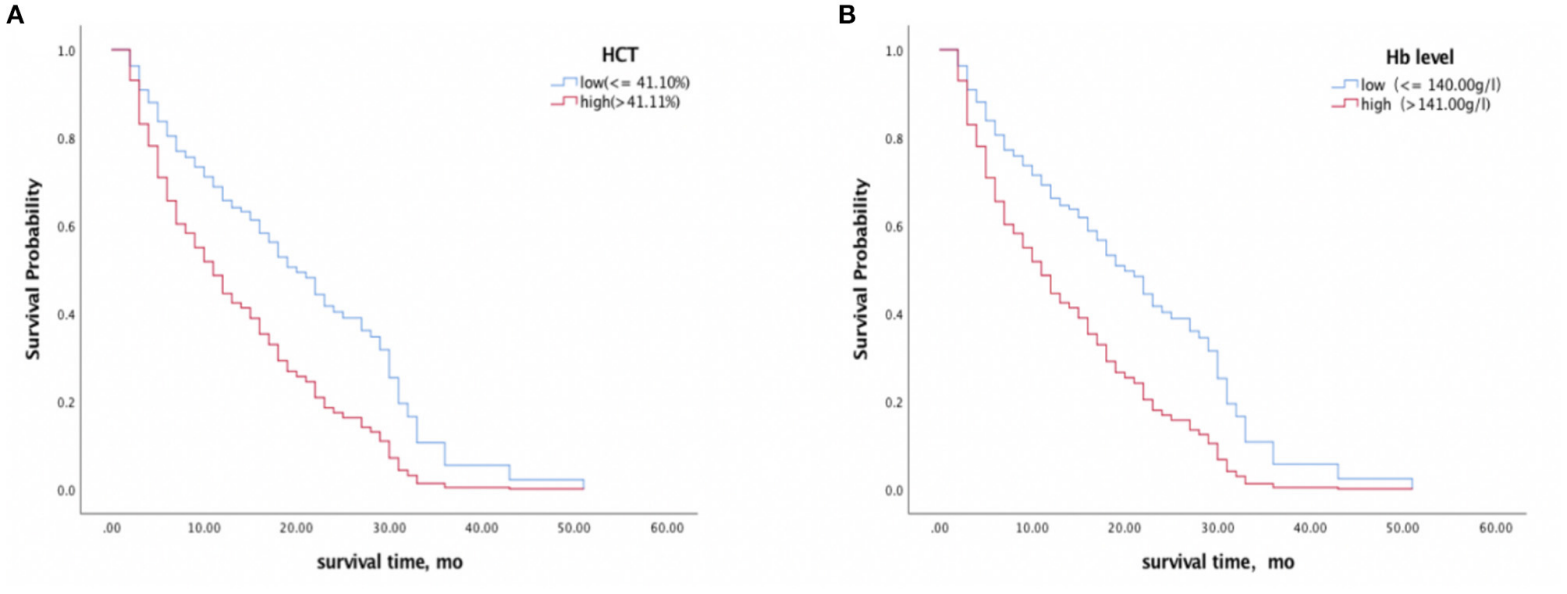
Survival analyses of CJD patients with high vs. low HCT **(A)** and Hb **(B)** levels. A median split was conducted on HCT (41.10%) and Hb (140.00 g/l) levels. Higher baseline Hb and HCT levels were associated with a shorter survival time.

Each erythrocyte index was assessed as a predictor of survival time (Table 3). Higher baseline Hb and HCT were associated with shorter survival time, with HR values of 1.710 (95% CI 1.124–2.600, *p* = 0.012) and 1.689 (95% CI 1.112–2.565, *p* = 0.014) respectively, and this association remained significant after controlling for age, sex, and Barthel Index (Hb, HR 1.959, 95% CI 1.232–3.116, *p* = 0.004; HCT, HR 1.927, 95%CI 1.234–3.010, *p* = 0.004) (Figure 3). In contrast, RBC, MCV, MCH, MCHC, and RDW did not significantly correlate with survival with or without adjustment for the confounding effects of covariates.

## Discussion

We investigated the association between red cell indices and survival times in 125 patients with CJD. The results showed that higher Hb and HCT levels were associated with shorter survival times in patients with CJD. This was the first study to demonstrate the relationship between peripheral blood erythrocytes and disease duration in patients with CJD, and suggests that Hb and HCT may be potential prognostic indicators.

The median survival time in our study was 12 months (range: 2.0–51.0 months) and 55.7% of the patients in our study died within one year of onset of the first symptom. Although disease progression was slower in our study than that in other studies (22, 23), a survey performed by the Japanese CJD surveillance program showed a longer survival time, with a mean disease course of 17.4 months and 46.0% mortality within one year (24). Our study indicated that younger age was associated with longer disease duration, which was consistent with the results of previous studies (3, 22, 25). As CJD is irreversible and fatal, the severity of the disease at baseline may predict prognosis. We found that lower baseline levels of function, as measured by Barthel Index, predicted shorter survival time (26).

In our study, Hb and HCT correlated with survival time in patients with CJD. Previous studies have found that both lower and higher hemoglobin levels were associated with an increased risk for developing AD, and worse cognitive performance and faster cognitive decline, which suggests that erythrocytic hemopoiesis may be important in neurodegenerative processes (11, 27). Some studies have focused on the effects of PrP^C^ on homeostatic processes. Miele et al. (16) found a connection between prion pathogenesis and peripheral erythropoiesis through downregulation of the AHSP. Subsequent studies found that HBA2 was downregulated in cattle infected with atypical Bovine Spongiform Encephalopathy infected cattle, and reduced expression of several other erythroid genes, Kell, GPA, band 3, and ankyrin were observed in mice with prion disease (15, 28). These studies suggested that there was an association between erythrocytes and prion disease. The mechanisms linking Hb levels to disease duration in CJD patients have not been well-characterized, but upregulation of Hb has been discovered in sCJD brains, suggesting that it might be a compensatory mechanism of survival neurons (29). We hypothesized that downregulation of erythroid genes in patients with CJD may result in reduced Hb synthesis, but compensatory mechanisms may result in increased Hb synthesis in peripheral blood. Therefore, high levels of Hb may reflect the severity of prion pathology. In addition, high Hb and HCT levels may aggravate the disease via ischemic and hypoxic mechanisms, as reported in studies of AD and chronic obstructive pulmonary disease (associated with high Hb levels) (11, 30). Although mechanisms are unclear, it is possible that Hb and HCT could be associated with survival time in CJD. Furthermore, our results suggested that erythrocyte might be a potential therapeutic target. However, further studies are needed to verify our research results and to explore the mechanisms by which hematopoiesis and prion disease interact.

The Barthel Index from the date of sampling did not correlate with red cell indices, which indicated that red cell indices did not reflect disease severity in patients with CJD. This may also explain poor accuracy of the Barthel Index. Other assessment measures, such as the Medical Research Council (MRC) Prion Disease Rating Scale, could be used in further studies. 14-3-3 protein in CSF is considered to be a reliable biomarker for the diagnosis of CJD and relates to extensive brain tissue damage (31). Our result that the positive rate of 14-3-3 test was similar to those researches based on the Chinese population, but was much lower than those raised in other countries (32–35). We explain such a difference might be related to ethnic differences or most of the patients we recruit in the early disease stage. In addition, no significant correlation was found between 14-3-3 protein and erythrocyte indices, indicating that erythrocyte indices could not reflect acute neuronal destruction. Considering the limitation of the western blot technique in 14-3-3 evaluation, quantitative measurement by ELISA might be recommended in further study.

Our study had some limitations. The majority of patients with CJD recruited for our study were probable diagnoses without a pathological diagnosis. Although we were able to find an association between Hb, HCT, and survival time in CJD, we were unable to determine the underlying mechanisms. In the present study, we did not analyze PRNP codon 129 genotypes, which have been found to correlate with survival time. However, MM type accounted for 97.0–100.0% of all sCJD patients, according to previous studies performed in China (36–38). Therefore, the different patterns of codon 129 genotypes could not explain the results. Also, due to the multiple risk factors that may be associated with Hb, HCT, and survival time in CJD, we were not able to adjust for other factors that may be associated with these items.

## Conclusions

This was the first study to investigate the relationship between red cell indices and survival time in patients with CJD patients, and our findings suggested that high Hb and HCT levels were associated with short disease duration. As red cell indices are frequently measured in current clinical practice, these results may have important value for estimating survival time in patients with CJD. However, the results should be interpreted with caution, and further studies are needed to confirm the results and explore the underlying mechanisms.

## Data Availability Statement

The original contributions presented in the study are included in the article/supplementary material, further inquiries can be directed to the corresponding author/s.

## Ethics Statement

The studies involving human participants were reviewed and approved by the Ethics Committee of Xuanwu Hospital, Capital Medical University. The patients/participants provided their written informed consent to participate in this study.

## Author Contributions

YK: original draft, data curation, and data analysis. ZC and JZ: review and editing. LW: supervision. All authors contributed to the article and approved the submitted version.

## Funding

This work was supported by the Ministry of Science and Technology of China (Grant No. 2019YFC0118600), the National Natural Science Foundation of China (Grant No. 81971011), and the Beijing Municipal Science and Technology Committee (Grant Nos. D171100008217005 and 7202060).

## Conflict of Interest

The authors declare that the research was conducted in the absence of any commercial or financial relationships that could be construed as a potential conflict of interest.

## Publisher's Note

All claims expressed in this article are solely those of the authors and do not necessarily represent those of their affiliated organizations, or those of the publisher, the editors and the reviewers. Any product that may be evaluated in this article, or claim that may be made by its manufacturer, is not guaranteed or endorsed by the publisher.
